# Prompt antimicrobial therapy and source control on survival and defervescence of adults with bacteraemia in the emergency department: the faster, the better

**DOI:** 10.1186/s13054-024-04963-7

**Published:** 2024-05-24

**Authors:** Ching-Chi Lee, Po-Lin Chen, Ching-Yu Ho, Ming-Yuan Hong, Yuan-Pin Hung, Wen-Chien Ko

**Affiliations:** 1 Clinical Medical Research Center, National Cheng Kung University Hospital, College of Medicine, National Cheng Kung University, No. 138, Sheng Li Road, 70403 Tainan, Taiwan; 2 Division of Infectious Disease, Departments of Internal Medicine, National Cheng Kung University Hospital, College of Medicine, National Cheng Kung University, No. 138, Sheng Li Road, 70403 Tainan, Taiwan; 3 Department of Adult Critical Care Medicine, Tainan Sin-Lau Hospital, No.57, Sec. 1, Dongmen Road, East Dist., Tainan, 70142 Taiwan; 4 Department of Nursing, National Tainan Junior College of Nursing, Tainan, Taiwan; 5 Departments of Emergency Medicine, National Cheng Kung University Hospital, College of Medicine, National Cheng Kung University, No. 138, Sheng Li Road, 70403 Tainan, Taiwan; 6 Department of Medicine, Medical College, National Cheng Kung University, No. 138, Sheng Li Road, 70403 Tainan, Taiwan; 7 Department of Internal Medicine, Tainan Hospital, Ministry of Health and Welfare, No. 125, Jhongshan Rd., West Central Dist., Tainan City, Taiwan

**Keywords:** Empirical, Antimicrobial therapy, Source control, Bloodstream infection, Defervescence, Mortality

## Abstract

**Background:**

Bacteraemia is a critical condition that generally leads to substantial morbidity and mortality. It is unclear whether delayed antimicrobial therapy (and/or source control) has a prognostic or defervescence effect on patients with source-control-required (ScR) or unrequired (ScU) bacteraemia.

**Methods:**

The multicenter cohort included treatment-naïve adults with bacteraemia in the emergency department. Clinical information was retrospectively obtained and etiologic pathogens were prospectively restored to accurately determine the time-to-appropriate antibiotic (TtAa). The association between TtAa or time-to-source control (TtSc, for ScR bacteraemia) and 30-day crude mortality or delayed defervescence were respectively studied by adjusting independent determinants of mortality or delayed defervescence, recognised by a logistic regression model.

**Results:**

Of the total 5477 patients, each hour of TtAa delay was associated with an average increase of 0.2% (adjusted odds ratio [AOR], 1.002; *P* < 0.001) and 0.3% (AOR 1.003; *P* < 0.001) in mortality rates for patients having ScU (3953 patients) and ScR (1524) bacteraemia, respectively. Notably, these AORs were augmented to 0.4% and 0.5% for critically ill individuals. For patients experiencing ScR bacteraemia, each hour of TtSc delay was significantly associated with an average increase of 0.31% and 0.33% in mortality rates for overall and critically ill individuals, respectively. For febrile patients, each additional hour of TtAa was significantly associated with an average 0.2% and 0.3% increase in the proportion of delayed defervescence for ScU (3085 patients) and ScR (1266) bacteraemia, respectively, and 0.5% and 0.9% for critically ill individuals. For 1266 febrile patients with ScR bacteraemia, each hour of TtSc delay respectively was significantly associated with an average increase of 0.3% and 0.4% in mortality rates for the overall population and those with critical illness.

**Conclusions:**

Regardless of the need for source control in cases of bacteraemia, there seems to be a significant association between the prompt administration of appropriate antimicrobials and both a favourable prognosis and rapid defervescence, particularly among critically ill patients. For ScR bacteraemia, delayed source control has been identified as a determinant of unfavourable prognosis and delayed defervescence. Moreover, this association with patient survival and the speed of defervescence appears to be augmented among critically ill patients.

**Supplementary Information:**

The online version contains supplementary material available at 10.1186/s13054-024-04963-7.

## Background

Bloodstream infections generally result in substantial morbidity and mortality, and thus are indicative of substantial healthcare costs, despite recent improvements in critical care and antimicrobial strategies [1, 2]. To achieve the improved outcomes of individuals with severe sepsis or septic shock, the Surviving Sepsis Campaign (SSC) recommends that all patients be assessed as soon as possible to identify sites of infections amenable to source control [3]. Although clinicians frequently encounter cases of bacteraemia complicated by abscesses or obstructive foci, there is a lack of evidence regarding the effect of time to source control on the prognosis of such patients. Moreover, to reduce hospitalisation periods and achieve the improved quality of care, it is necessary to understand the clinical condition (such as the timing of defervescence) in response to adequate source control for patients experiencing source-control-required (ScR) bacteraemia. However, the benefit of prompt source control on defervescence in cases of ScR bacteraemia remains unclear. Therefore, we hypothesised that prompt source control is a crucial determinant of improved outcomes and rapid defervescence in individuals with ScR bloodstream infections, particularly those initially presenting with critical illness.

Although etiologic pathogens and their susceptibilities may differ across areas [4], the appropriateness of empirical antimicrobials administered has been shown to be prognostically advantageous in various types of bloodstream infections, such as their episodes on general wards [5] or intensive care units (ICUs)[6], community-onset bacteraemia [7, 8], and bacteraemia caused by carbapenem-resistant Enterobacterales [9]. Another challenge for clinicians in treating bacteraemia patients is the rapid identification of bacteraemia sources and the prompt recognition of the need for source control. However, the investigation comparing the prognostic impact of delayed administration of appropriate antibiotics on patients with ScR bacteraemia and those without the need of source control remains limited. Furthermore, to achieve improved prognoses, faster administration of appropriate antimicrobials is essential for more severe episodes of bacteraemia. [7, 8]. Therefore, we hypothesised that appropriate empirical therapy would predominantly be more advantageous for improving short-term prognoses among more critically ill patients, regardless of whether the bacteraemia was source-control required or unrequired. The present study aimed to investigate the association of delayed antimicrobial therapy and/or source control and short-term mortality and/or defervescence in individuals with bacteraemia, specifically categorizing them into two groups: ScR and source-control unrequired (ScU) bacteraemia.

## Methods

### Study design

This multicenter cohort study was conducted over a period of six years, from January 2016 to December 2021, in the emergency departments (EDs) of two teaching hospitals (380 and 460 beds) and one university-affiliated medical center (1193 beds) in southern Taiwan. The target population in this study comprised treatment-naïve adults (aged ≥ 18 years) experiencing bacteraemia in the ED to establish the Southern Taiwan ED BloodStream Infection (STEDBSI) cohort. The study evaluated two exposures assessed by hours: time-to-appropriate antibiotic (TtAa) and time-to-source control (TtSc). The study outcomes included crude mortality within 30 days after ED arrival (i.e., the onset of bacteraemia) and the time of defervescence. The study received approval from the Institutional Review Board of all study hospitals. The ethics committee waived the requirement for written the informed consent from each participant, considering the nature of the retrospective study. The study adhered to the guidelines outlined in the Strengthening the Reporting of Observational Studies in Epidemiology (STROBE) statement (Supplementary Table 1).

### Patient selections

Of the medical and surgical ED visits, those sampled with blood cultures in the ED were screened for bacterial growth using an electronic medical record. Of adults with bacterial growth, those with contaminated sampling of cultures or those treated with any antimicrobial prior to visiting the ED were excluded. Moreover, patients lacking clinical information, such as image studies that made confirmation for infection sources amenable to control, the time of appropriate source control or antimicrobial therapy, or uncertain fatality prior to the study endpoint (i.e., loss to follow-up), were also excluded. Subgroup analysis focused on patients initially presenting with fever to assess the association of study exposures and defervescence. Therefore, patients who were afebrile or hypothermic during their ED stay, regularly taking steroids or antipyretics during antimicrobial therapy, experiencing drug anaphylaxis, tumor fever, adrenal insufficiency, or hospital-onset infections, or those with an uncertain date of defervescence were excluded from the subgroup analysis.

### Data collection

A predetermined record form was adopted to manually capture patient demographics and clinical characteristics during the ED stay, in terms of patient demographics (age, gender), bacteraemia severity (Pitt bacteraemia score [PBS]), comorbid severity (McCabe classification), comorbid types, laboratory data, and image studies. Further information regarding hospitalisation and managements, such as durations and types of antimicrobial therapy, imaging studies, microbiological results, bacteraemia sources, dates and types of surgical or radiologic interventions, the time of defervescence, and patient prognoses were also manually obtained by reviewing the electronic medical record. For eligible patients with missing data, a normal value was inserted for continuous variables and a negative value was inserted for categorical variables. The study endpoints included 30-day mortality in all eligible patients and the time of defervescence in the febrile subgroup. All clinical data were independently captured by a board-certified infectious-disease (ID) physician and an ED clinician who had a full experience in reviewing medical charts. Both were blind to the hypotheses and aim of the current study. Discrepancies in data capturing were resolved by conducting face-to-face discussions between the data abstracters.

### Sampling of blood cultures and microbiological methods

In the ED, blood sampling was performed by nurses or doctors. Two sets of blood cultures were obtained from different peripheral veins or arteries, ensuring a minimum of 30 minutes between each sampling. Each set of blood cultures typically consisted of one bottle for aerobic culture and another for anaerobic culture, with approximately 10 mL of blood collected in each bottle. The bottles containing blood cultures were promptly placed in a BACTEC 9240 instrument (Becton Dickinson Diagnostic Systems, Sparks, MD, USA) and incubated for five days at 35ºC. To identify the causative bacteria, matrix-assisted laser desorption ionization time-of-flight mass spectrometry was used, and the identified bacteria were prospectively stored in glycerol stocks at −80 °C for potential susceptibility testing in the future. The susceptibilities were tested in accordance with the 2023 CLSI standard [10], using the agar dilution method for anaerobes and the disk diffusion method for aerobes. The antibiotics assessed for Gram-negative aerobes included levofloxacin, moxifloxacin, ampicillin/sulbactam, piperacillin/tazobactam, imipenem, ertapenem, cefepime, ceftazidime, cefotaxime, cefuroxime, and cefazolin. Ampicillin and penicillin were tested for enterococci and streptococci, respectively. Anaerobes were evaluated for susceptibility to moxifloxacin, piperacillin/tazobactam, ampicillin/sulbactam, and metronidazole. To ensure the appropriate timing of antibiotic administration for each patient, susceptibility was performed for the specific antibiotic indicated, provided that the initial susceptibility panel did not already include empirical antibiotics.

### Definitions

The term "bacteraemia" is the presence of bacteria in blood cultures obtained from peripheral or central venipuncture, excluding cultures that are contaminated [2]. Blood cultures containing potentially contaminating pathogens, such as Bacillus species, micrococci, Propionibacterium species, Gram-positive bacilli, and coagulase-negative staphylococci, were identified as contaminated based on previous criteria [11]. More than one microbial species isolated from a single bacteraemia episode was polymicrobial bacteraemia.

As previously described [8, 9], antimicrobial treatment was considered appropriate if it included adequate dosage and route of administration, and if it demonstrated *in vitro* activity against all identified pathogens causing bacteremia. Treatment that did not meet these criteria or was not administered was defined as inappropriate treatment. The time gap (measured in hours) between the ED triage (i.e., ED arrival) and appropriate administration of antimicrobials was referred to the TtAa.

Based on the isolation of pathogens and/or clinical diagnoses, in line with established concepts [12, 13], a patient was allocated to one of the primary bacteraemia sources. As previously suggested [14, 15], the need of source control and the appropriateness of specific percutaneous or surgical control, such as the drainage of infected fluid accumulation, drainage of abscesses or obstructive tracts, removal of infected devices, debridement of infected necrotic tissue, and definitive control of sources underwent microbial contamination, were jointly determined by one board-certified ID and another ED physician (as part of the data capturing process), The source control techniques enrolled in our study were listed in detail in Appendix S1. Based on whether the requirement for source control techniques was met or not, the eligible patients were categorised into the patient groups of ScU and ScR bacteraemia. The time gap in hours from the ED triage to appropriate source control was assessed as the TtSc. Based on the presence of abscesses recognised in image studies, ScR bacteraemia was categorised into the abscess and non-abscess groups. The types of abscesses identified in varied bacteraemia sources were listed in detail in Appendix S2.

Temporal body temperature (BT) was measured routinely every four hours in the ED and general wards and every two hours in ICUs. If fever or hypothermia occurred more than once in one patient, the extreme BT was recorded. Hypothermia was defined as a temporal BT of ≤ 36.0 °C. Defervescence was defined as a state of being afebrile, with a tympanic BT consistently below 37.0 °C for at least 24 hours [8, 16]. The period from the ED triage to the onset of defervescence was defined as the ED-to-defervescence period. Patients with an ED-to-defervescence exceeding 75% of the total lengths of ED-to-defervescence within the febrile subgroup were regarded as individuals with delayed defervescence. The severity of bacteraemia at onset was graded by a validated scoring algorithm (PBS) including body temperatures, cardiac arrest, receipt of mechanical ventilation, blood pressure or usage of vasopressor agents, and mental status [17]. A patient with a PBS of ≥ 4 points was categorised as critically ill [17]. Comorbidities were defined as previously described [18], and the comorbid prognosis was assessed by an established classification (McCabe classification) [19]. Crude mortality was equated with death from all causes.

### Statistical analyses

Statistical analyses were performed using the Statistical Package for the Social Sciences for Windows (Version 23.0; Chicago, IL, USA). Categorical variables, expressed as numbers and percentages, were compared by the *Chi-*square method or Fisher’s exact test (if expected values < 5). Continue variables. expressed as medians and interquartile ranges (IQRs), were assessed using the independent t test or Mann–Whitney U test. Spearman’s correlation was used to analyze the linear-by-linear association of the TtAa or TtSc periods with 30-day crude mortality rates for all eligible patients. For febrile patients, Spearman’s correlation was used to study the linear-by-linear association of the TtAa or TtSc period with the ED-to-defervescence.

To identify the independent predictors of 30-day crude mortality or delayed defervescence, variables with a univariate *P* value < 0.05 were included in a stepwise and backward model of logistic regression. To control for confounding factors, the association of TtAa or TtSc delay (in hours) and 30-day mortality was investigated after adjusting for all independent predictors of 30-day crude mortality. Similarly, the association of TtAa or TtSc delay (in hours) and delayed defervescence was investigated after adjusting for independent predictors of delayed defervescence identified by logistic regression. A *P* value < 0.05 was considered statistically significant.

## Results

### Patient demographics and etiologic pathogens in the overall cohort

During the 6-year period, a cohort of 5477 patients was studied, comprising 3953 (72.2%) with ScU bacteraemia and 1524 (27.8%) with ScR bacteraemia, as determined by the inclusion and exclusion criteria (Supplemental Fig. 1). The proportion of patients with missing data for each variable of interest in our study was listed in Supplemental Fig. 2. Of the overall cohort, the median (IQR) age of the patients was 70 (57–80) years, 2862 (52.3%) were male, and their median (IQR) of TtAa 2.0 (1.0–9.4) hours. The median (IQR) ED and hospital stay were 16.2 (6.0–27.5) hours and 10.0 (6.0–18.0) days, respectively. Most patients (5146, 94.0%) were hospitalised through the ED, 178 (3.2%) died in the ED, and only 153 (2.8%) were discharged from the ED and followed up in outpatient clinics. The proportion of critically ill patients (PBS ≥ 4) in the ED and 30-day crude mortality rate were 21.3% (1164 patients) and 15.5% (849), respectively.

Polymicrobial bacteraemia occurred in 481 episodes, with a total of 6253 causative microorganisms collected from the cohort patients. The distribution of etiologic pathogens and their susceptibilities were detailed in Appendix S3. A total of 494 patients received combinative antimicrobial therapy, resulting in the administration of 6453 antibiotics as empirical agents. The proportion of antimicrobials empirically administered was listed in Appendix S4.

Among 1524 patients with ScR bacteraemia, the median (IQR) of TtSc was 72 (20–256) hours. The most common technique for source control was the abscess incision and debridement (354 patients, 23.2%), followed by percutaneous or internal biliary drainage (329, 21.6%), percutaneous abdominal drainage (244, 14.7%), percutaneous nephrostomy (218, 14.3%), fasciotomy or fasciectomy (108, 7.1%), hemicolectomy or colostomy (97, 6.4%), percutaneous chest tube drainage (55, 3.6%), appendectomy (30, 2.0%), vascular surgery (28, 1.8%), cholecystectomy (21, 1.4%), bronchus and lung surgery (15, 1.0%), and valvular heart surgery (8, 0.5%). Seventeen patients (1.1%) did not undergo any source control prior to their rapid fatality.

### A comparison of ScU and ScR bacteraemia in the overall cohort

For overall patients, a univariate analysis (Table 1) was conducted to compare clinical demonstrations and crude mortality rates between patients with ScU and ScR bacteraemia. Male, critical illness (PBS ≥ 4), bacteraemia caused by skin and soft-tissue, biliary tract, intraabdominal, or bone and joint infections, polymicrobial bacteraemia, etiologic pathogens of *K. pneumoniae*, *S. aureus*, anaerobes, or enterococci, and comorbid diabetes mellitus were more frequently observed in patients with ScR bacteraemia. Conversely, a lower proportion of nursing-home residents, bacteraemia caused by urinary tract or low respiratory infections, etiologic pathogens of *E. coli*, streptococci, or *Pseudomonas* species, fatal comorbidities (by McCabe classification), and comorbidities of malignancies or neurological diseases were observed in patients with ScR bacteraemia. Of note, no significant difference was exhibited in the TtAa period and 15-day or 30-day crude mortality rates between the two patient groups.

**Table 1:** Clinical manifestations and patient outcomes between source-control-required (ScR) and unrequired (ScU) bacteraemia*

Variable	Patient number (%)		*P* value
	ScR, n = 1524	ScU, n = 3953	
Patient demography			
Age, year, median (IQR)	69.0 (57.0–79.0)	70.0 (57.0–80.0)	0.09
Gender, male	871 (57.2)	1991 (50.4)	< 0.001
Nursing-home resident	51 (3.3)	223 (5.6)	< 0.001
Body mass index, kg/m^2^, median (IQR)	23.1 (20.0–26.4)	22.9 (19.7–26.2)	0.20
Time-to-appropriate antibiotic, hour, median (IQR)	2.0 (1.0–8.1)	2.0 (1.0–10.0)	0.06
Pitt bacteraemia score ≥ 4 at onset	354 (23.2)	810 (20.5)	0.03
Major bacteraemia source			
Skin and soft-tissue	362 (23.8)	258 (6.5)	< 0.001
Biliary tract	354 (23.2)	193 (4.9)	< 0.001
Urinary tract	257 (16.9)	1528 (38.7)	< 0.001
Intra-abdominal	240 (15.7)	425 (10.8)	< 0.001
Bone and joint	162 (10.6)	65 (1.6)	< 0.001
Low respiratory tract	125 (8.2)	821 (20.8)	< 0.001
Polymicrobial bacteraemia	191 (12.5)	290 (7.3)	< 0.001
Major etiologic pathogen			
*Escherichia coli*	448 (29.4)	1570 (39.7)	< 0.001
*Klebsiella pneumoniae*	487 (32.0)	762 (19.3)	< 0.001
*Staphylococcus aureus*	229 (15.0)	420 (10.6)	< 0.001
Streptococci	152 (10.0)	583 (14.7)	< 0.001
Anaerobes	124 (8.1)	103 (2.6)	< 0.001
Enterococci	62 (4.1)	103 (2.6)	0.005
*Pseudomonas* species	28 (1.8)	149 (3.8)	< 0.001
Fatal comorbidity (McCabe classification)	334 (21.9)	1071 (27.1)	< 0.001
Major comorbidity			
Cardiovascular disease	825 (54.1)	2154 (54.5)	0.81
Diabetes mellitus	652 (42.8)	1496 (37.8)	0.001
Malignancy	219 (14.4)	668 (16.9)	0.02
Chronic kidney disease	330 (21.7)	814 (20.6)	0.39
Neurological disease	308 (20.2)	976 (24.7)	< 0.001
Liver cirrhosis	158 (10.4)	489 (12.4)	0.04
Crude mortality rates			
3-day	98 (6.4)	285 (7.2)	0.31
15-day	187 (12.3)	469 (11.9)	0.68
30-day	254 (16.7)	595 (15.1)	0.14

ED = emergency department; IQR = interquartile range. *Boldface indicates statistical significance with a *P* value of < 0.05 under the univariate analysis and data are number (%) of patients unless otherwise stated

### TtAa and TtSc on mortality in ScU and ScR bacteraemia

**Fig. 1:**
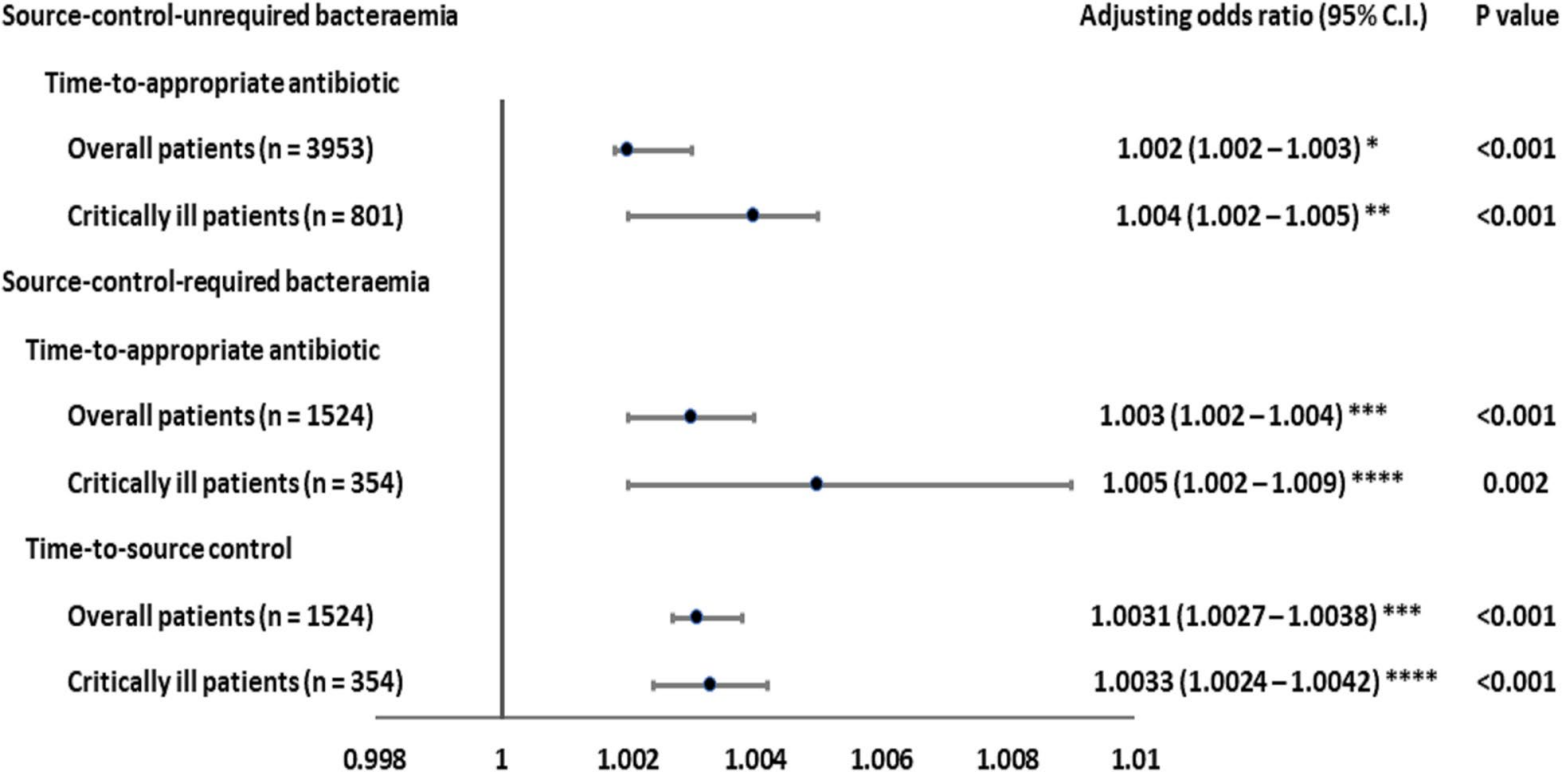
The association of the time-to-appropriate antibiotic or time-to-source control and 30-day crude mortality. *Adjusted for eight independent predictors of 30-day mortality in overall patients with source-control-unrequired bacteraemia (Supplemental Table 2). **Adjusted for five independent predictors of 30-day mortality in critically ill patients with source-control-unrequired bacteraemia (Supplemental Table 4). ***Adjusted for 13 independent predictors of 30-day mortality in overall patients with source-control-required bacteraemia (Supplemental Table 3). ****Adjusted for five independent predictors of 30-day mortality in critically ill patients with source-control-required bacteraemia (Supplemental Table 4)

A positive linear-by-linear association of TtAa and 30-day mortality rates (all, *ρ* = 1.00, *P* = 1.00) in the overall and critically ill patients with ScU (Supplemental Fig. 3A) or ScR (Supplemental Fig. 3B) bacteraemia was exhibited, respectively. In further analyses (Fig. 1), each hour of TtAa delay was respectively associated with an average increase of 0.2% (adjusted odds ratio [AOR], 1.002; *P* < 0.001) and 0.4% (AOR 1.004; *P* < 0.001) in 30-day crude mortality rates for the overall and critically ill patients experiencing ScU bacteraemia, after adjusting for the independent predictors of 30-day mortality (Supplemental Tables 2 and 4). For patients experiencing ScR bacteraemia (Table 2), each hour of TtAa delay was, respectively, associated with an average increase of 0.3% (AOR 1.003; *P* < 0.001) and 0.5% (AOR 1.005; *P* = 0.002) in 30-day crude mortality rates for the overall and critically ill individuals, after adjusting the independent predictors of 30-day mortality (Supplemental Tables 3 and 4).

**Table 2:** Clinical manifestations and outcomes of febrile patients between source-control-required (ScR) and unrequired (ScU) bacteraemia*

Variable	Patient number (%)		*P* value
	ScR, n = 1266	ScU, n = 3085	
Patient demography			
Age, year, median (IQR)	68.0 (57.0–79.0)	69.0 (56.0–80.0)	1.00
Gender, male	723 (57.1)	1484 (48.1)	< 0.001
Nursing-home resident	39 (3.1)	139 (4.5)	0.03
Body mass index, kg/m^2^, median (IQR)	23.3 (20.4–26.6)	23.1 (20.1–26.2)	0.25
Time-to-appropriate antibiotic, hour, median (IQR)	2.0 (1.0–8.0)	2.0 (1.0–7.2)	0.63
ED-to-defervescence, day, median (IQR)	9.5 (5.0–20.0)	4.0 (3.0–8.0)	< 0.001
Pitt bacteraemia score ≥ 4 at onset	264 (20.9)	486 (15.8)	< 0.001
Major bacteraemia source			
Biliary tract	303 (23.9)	162 (5.3)	< 0.001
Skin and soft-tissue	286 (22.6)	218 (7.1)	< 0.001
Urinary tract	213 (16.8)	1320 (42.8)	< 0.001
Intra-abdominal	196 (15.5)	334 (10.8)	< 0.001
Bone and joint	125 (9.9)	49 (1.6)	< 0.001
Low respiratory tract	98 (7.7)	458 (14.8)	< 0.001
Polymicrobial bacteraemia	146 (11.5)	195 (6.3)	< 0.001
Major etiologic pathogen			
*Klebsiella pneumoniae*	429 (33.9)	593 (19.2)	< 0.001
*Escherichia coli*	371 (29.3)	1326 (43.0)	< 0.001
*Staphylococcus aureus*	174 (13.7)	283 (9.2)	< 0.001
Streptococci	124 (9.8)	439 (14.2)	< 0.001
Anaerobes	91 (7.2)	67 (2.2)	< 0.001
Enterococci	52 (4.1)	73 (2.4)	0.002
Pseudomonas species	19 (1.5)	111 (3.6)	< 0.001
Fatal comorbidity (McCabe classification)	252 (19.9)	756 (24.5)	0.001
Major comorbidity			
Cardiovascular disease	670 (52.9)	1666 (54.0)	0.52
Diabetes mellitus	551 (43.5)	1186 (38.4)	0.002
Chronic kidney disease	256 (20.2)	609 (19.7)	0.72
Neurological disease	245 (19.4)	703 (22.8)	0.01
Malignancy	166 (13.1)	470 (15.2)	0.07
Liver cirrhosis	131 (10.3)	360 (11.7)	0.21
Crude mortality rates			
3-day	53 (4.2)	127 (4.1)	0.92
15-day	118 (9.3)	226 (7.3)	0.03
30-day	172 (13.6)	301 (9.8)	< 0.001

ED = emergency department; IQR = interquartile range. *Boldface indicates statistical significance with a *P* value of < 0.05 under the univariate analysis and data are number (%) of patients unless otherwise stated

A positive linear-by-linear association of TtSc and 30-day mortality rates (all, *ρ* = 1.00, *P* = 0.01) was exhibited among overall and critically ill patients with ScR bacteraemia (Supplemental Fig. 3C). Furthermore (Fig. 1), each hour delay of TtSc was associated with an average increase of 0.31% (AOR 1.0031; *P* < 0.001) and 0.33% (AOR 1.0033; *P* = 0.002) in 30-day crude mortality rates for the overall and critically ill patients with ScR bacteraemia, respectively, after adjusting for the independent predictors of 30-day mortality (as shown in Supplemental Tables 3 and 4)

### A comparison of ScU and ScR bacteraemia in the febrile subgroup

For patients with initial febrile presentation, a univariate analysis was conducted to compare clinical variables and crude mortality rate between ScU and ScR bacteraemia (Table 2). Male, critical illness (PBS ≥ 4), bacteraemia caused by biliary tract, skin and soft-tissue, intra-abdominal, or bone and joint infections, polymicrobial bacteraemia, etiologic pathogens of *K. pneumoniae*, *S. aureus*, anaerobes, or enterococci, and comorbid diabetes mellitus were frequently observed in patients with ScR bacteraemia. Conversely, patients with ScR bacteraemia exhibited the lower proportions of nursing-home residents, bacteraemia caused by urinary tract or low respiratory infections, etiologic pathogens of *E. coli*, streptococci, or *Pseudomonas* species, fatal comorbidities, and comorbid neurological diseases, compared to those with ScU bacteraemia. Notably, patients with ScR bacteraemia had a longer period of ED-to-defervescence and a higher rate of 15- or 30-day crude mortality, compared with those with ScU bacteraemia.

### TtAa and TtSc on delayed defervescence in the febrile subgroup

**Fig. 2:**
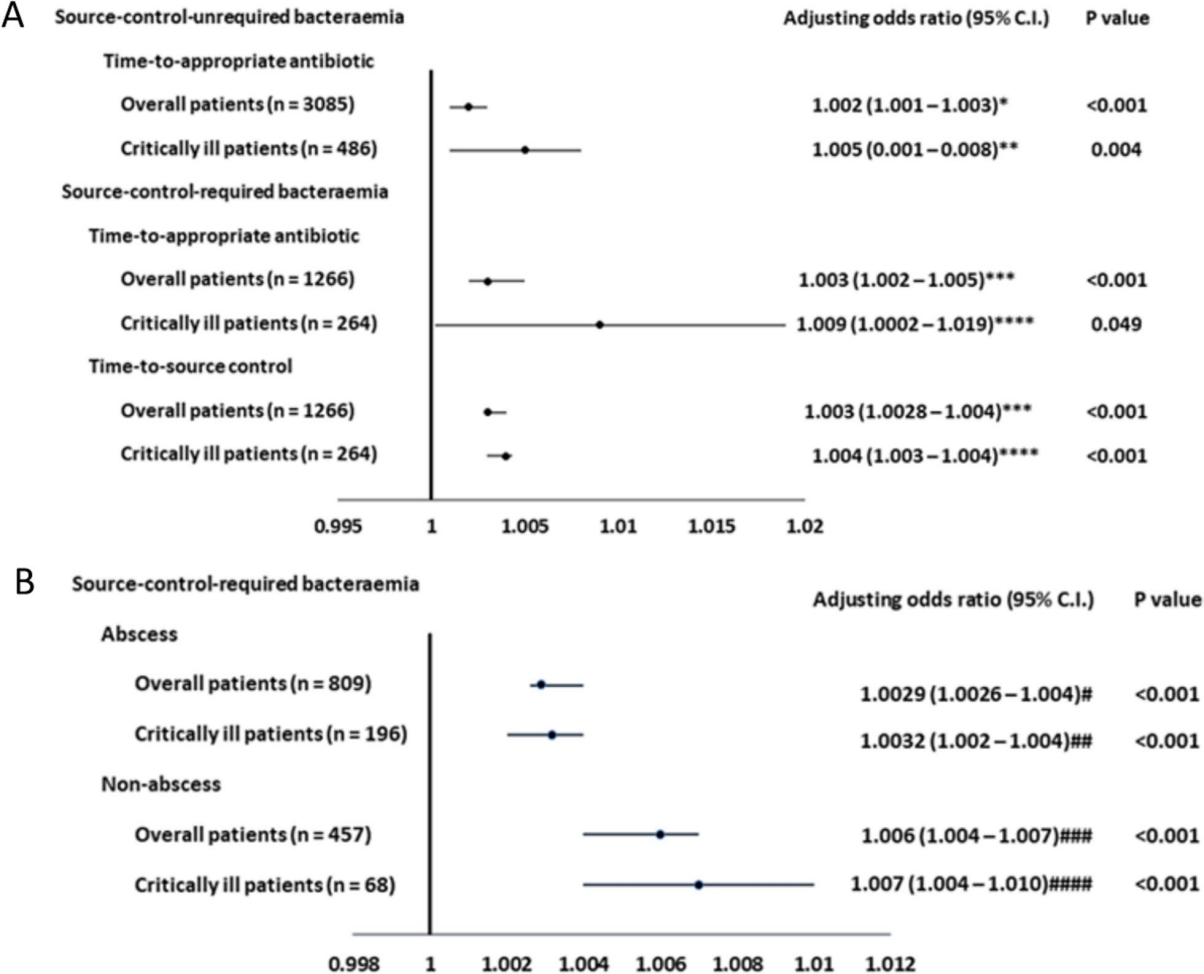
Associations between the time-to-appropriate antibiotic or time-to-source control and delayed defervescence among patients with source-control-unrequired (ScU) and required (ScR) bacteraemia (2A), and among those with ScR bacteraemia categorised into the abscess and non-abscess groups (2B)^§^. ^§^ The ED-to-defervescence of ≥ 8 and ≥ 20 days in source-control-unrequired bacteraemia and source-control-required bacteraemia was defined as delayed defervescence, respectively. *Adjusted for eight independent predictors of delayed defervescence in overall patients with source-control-unrequired bacteraemia (Supplemental Table 5). ** Adjusted for three independent predictors of delayed defervescence in critically ill patients with source-control-unrequired bacteraemia (Supplemental Table 7). *** Adjusted for ten independent predictors of delayed defervescence in overall patients with source-control-required bacteraemia (Supplemental Table 6). ****Adjusted for four independent predictors of delayed defervescence in critically ill patients with source-control-required bacteraemia (Supplemental Table 7). ^#^Adjusted for six independent predictors of delayed defervescence in patients with abscess occurrence (Supplemental Table 8). ^##^Adjusted for three independent predictors of delayed defervescence in critically ill patients with abscess occurrence (Supplemental Table 9) ^###^Adjusted for six independent predictors of delayed defervescence in patients without abscess occurrence (Supplemental Table 8). ^####^No independent predictor of delayed defervescence was adjusted in critically ill patients without abscess occurrence (Supplemental Table 9)

The range of the ED-to-defervescence for overall febrile patients was 1.2–61.0 days. In patients with ScU and ScR bacteraemia, the median (IQR) of the ED-to-defervescence was 4.0 (3.0–8.0) and 9.5 (5.0–20.0) days; and a patient with a delay of ≥ 8 and ≥ 20 days was grouped to have delayed defervescence in the two groups, respectively. A positive linear-by-linear association of TtAa and the proportion of patients with delayed defervescence in the overall (*ρ* = 1.00, *P* = 0.01) and critically ill (*ρ* = 0.90, *P* = 0.04) patients with ScU bacteraemia was exhibited, respectively (Supplemental Fig. 4A). Similarly, a positive linear-by-linear association of TtAa and such proportion in the overall and critically ill patients (all, *ρ* = 1.00, *P* = 0.01) with ScR bacteraemia was observed, respectively (Supplemental Fig. 4B). In further analyses (Fig. 2A), each additional hour of TtAa was, respectively, associated with an average increase of 0.2% (AOR 1.002; *P* < 0.001) and 0.5% (AOR 1.005; *P* = 0.004) in the proportion of patients with delayed defervescence for the overall and critically ill patients experiencing ScU bacteraemia, after adjusting for independent predictors of delayed defervescence (Supplemental Tables 5 and 7). For febrile patients having ScR bacteraemia (Fig. 2A), each additional hour of TtAa was, respectively, associated with an average increase of 0.3% (AOR 1.003; *P* < 0.001) and 0.9% (AOR 1.009; *P* = 0.049) in the proportion of patients with delayed defervescence for the overall and critically ill individuals, after adjusting for the independent predictors of delayed defervescence (Supplemental Tables 6 and 7).

A positive linear-by-linear association of TtSc and delayed defervescence was disclosed among the overall (*ρ* = 1.00, *P* = 0.01) and critically ill (*ρ* = 0.90, *P* = 0.04) patients with ScR bacteraemia, respectively (Supplemental Fig. 4C). Furthermore (Fig. 2A), each additional hour of TtSc was, respectively, associated with an average 0.3% (AOR 1.003; *P* < 0.001) and 0.4% (AOR 1.004; *P* < 0.001) increase in the proportion of patients with delayed defervescence among the overall and critically ill individuals with ScR bacteraemia, after adjusting for the independent predictors of delayed defervescence (Supplemental Tables 6 and 7).

### TtSc on defervescence in febrile ScR bacteraemia with or without abscess

Of 1266 patients experiencing ScR bacteraemia with initial febrile presentation, those with abscess formation accounted for 63.9% (809 patients). A significant difference of clinical characteristics and outcomes, in terms of patient demographics, bacteraemia sources, etiologic pathogens, comorbid types, bacteraemia severity, and crude mortality rates, between the abscess and non-abscess groups was demonstrated in Table 3. Notably, a period of TtSc and ED-to-defervescence in the abscess group was significant longer than that in the non-abscess group. As shown in Fig. 2B, each additional hour of TtSc was, respectively, associated with an average 0.29% (AOR 1.0029; *P* < 0.001) and 0.32% (AOR 1.0032; *P* < 0.001) increase in the proportion of patients with delayed defervescence among the overall and critically ill individuals in the abscess group, after adjusting for the independent predictors of delayed defervescence (Supplemental Tables 8 and 9). For febrile patients with ScR bacteraemia but without abscess formation (Fig. 2B), each hour delay of TtSc was associated with an average 0.6% (AOR 1.006; *P* < 0.001) and 0.7% (AOR 1.007; *P* < 0.001) increase in the proportion of patients with delayed defervescence among the overall and critically ill individuals, after respectively adjusting for the independent predictors of delayed defervescence (Supplemental Tables 8 and 9).

**Table 3:** Clinical manifestations and outcomes of febrile patients with source-control-required bacteraemia, categorised into the abscess and non-abscess groups*

Variable	Patient number (%)		*P* value
	Abscess, n = 809	Non-abscess, n = 457	
Patient demography			
Age, year, median (IQR)	64.0 (55.0–76.0)	74.0 (61.5–82.0)	< 0.001
Gender, male	485 (60.0)	238 (52.1)	0.007
Nursing-home resident	20 (2.5)	19 (4.2)	0.10
Time-to-source control, hour, median (IQR)	120.0 (23.0–189.0)	33.0 (15.6–102.0)	< 0.001
Time-to-appropriate antibiotic, hour, median (IQR)	2.0 (1.0–7.4)	2.9 (1.0–18.1)	0.02
ED-to-defervescence, day, median (IQR)	6.0 (14.0–41.0)	6.0 (4.0–12.0)	< 0.001
Pitt bacteraemia score ≥ 4 at onset	196 (24.2)	68 (14.9)	< 0.001
Major bacteraemia source			
Skin and soft-tissue	262 (32.4)	24 (5.3)	< 0.001
Liver abscess	194 (24.0)	0 (0)	< 0.001
Intra-abdominal	154 (19.0)	42 (9.2)	< 0.001
Bone and joint	99 (12.2)	26 (5.7)	< 0.001
Low respiratory tract	94 (11.6)	4 (0.9)	< 0.001
Urinary tract	68 (8.4)	145 (31.7)	< 0.001
Biliary tract	21 (2.6)	282 (61.7)	< 0.001
Polymicrobial bacteraemia	90 (11.1)	56 (12.3)	0.55
Major etiologic pathogen			
*Klebsiella pneumoniae*	296 (36.6)	133 (29.1)	0.007
*Staphylococcus aureus*	159 (19.7)	15 (3.3)	< 0.001
*Escherichia coli*	131 (16.2)	240 (52.5)	< 0.001
Streptococci	104 (12.9)	20 (4.4)	< 0.001
Anaerobes	78 (9.6)	13 (2.8)	< 0.001
Enterococci	25 (3.1)	27 (5.9)	0.02
Fatal comorbidity (McCabe classification)	162 (20.0)	90 (19.7)	0.89
Major comorbidity			
Cardiovascular disease	417 (51.5)	253 (55.4)	0.19
Diabetes mellitus	382 (47.2)	169 (37.0)	< 0.001
Chronic kidney disease	181 (22.4)	75 (16.4)	0.01
Neurological disease	148 (18.3)	97 (21.2)	0.21
Malignancy	89 (11.0)	77 (16.8)	0.003
Liver cirrhosis	95 (11.7)	36 (7.9)	0.03
Crude mortality rates			
3-day	42 (5.2)	11 (2.4)	0.02
15-day	91 (11.2)	27 (5.9)	0.002
30-day	135 (16.7)	37 (8.1)	< 0.001

ED = emergency department; IQR = interquartile range. *Boldface indicates statistical significance with a *P* value of < 0.05 under the univariate analysis and data are number (%) of patients unless otherwise stated

## Discussion

Previously, there was little consensus regarding the potential advantage of prompt administration of appropriate antimicrobials in bloodstream infections. Early studies suggested that such treatment had no effect [20, 21], but recent investigations involving large cohorts consistently demonstrate that it significantly reduces fatality in diverse patient populations [5, 6, 9]. In conformity with these updated findings [5, 6, 9], our study specifically examined patients with ScR or ScU bacteremia and found that delayed administration of appropriate antimicrobials was associated with unfavourable prognosis in both patients. Notably, corresponding with current knowledge suggesting that the faster administration of appropriate antimicrobials is essential for more severe episodes of sepsis [3] or bacteraemia [7, 8], our cohort demonstrated that the association between delayed administration of appropriate antimicrobials and short-term outcomes seem to be augmented in critically ill individuals, irrespective of whether their bacteraemia was source-control unrequired or required.

Source control aims to eliminate infection sources, control contamination, and restore anatomy and function [22]. Clinically, this involves draining infected fluids or abscesses, debriding infected or necrotic soft tissues, removing foreign bodies or infected devices, and correcting anatomic derangement that causes microbial contamination [23]. For patients experiencing severe sepsis and septic shock, numerous investigations have indicated that source control is a crucial determinant of patient outcomes [22–24]. Therefore, the updated SSC guideline recommends prompt evaluation for the presence of complicated infections and expeditious implementation of source control [3]. Consistent with the view that source control is necessary for severely ill patients [22–24] and intra-abdominal infections [25], our cohort found that the timing of source control was a crucial determinant of short-term mortality in patients with ScR bacteraemia. We believe this findings are rightfully reasonable because bacteraemia is generally regarded as a systemic infection and a life-threatening disease that causes substantial mortality and morbidity [1, 2]. In addition, bacterial load in infectious foci is the primary driver of septic organ dysfunction, and the appropriateness of source control could substantially result in the rapid clearance of etiologic pathogens and reducing the systemic inflammatory response [26]. It is our belief that that the association of delayed source control and short-term prognoses seem to be more pronounced in patients with ScR bacteraemia who initially presented with the critical illness. regardless of whether their bacteraemia originated from abscesses or not.

To shorten the length of hospitalisation, the timing of defervescence has been assessed as a crucial outcome in response to antimicrobial therapy in previous investigations detailing bloodstream infections [8, 16, 27]. Consistent with these studies [8, 16, 27], our cohort reasonably indicted the association between the defervescence timing and the appropriateness of empirical antimicrobial therapy among both febrile adults experiencing ScR and ScU bacteraemia. Because the appropriateness of source control could substantially lead to rapid clearance of etiologic pathogens and fast diminishing of systemic inflammation [26], early defervescence resulted from prompt source control were expected in bacteraemic patients. Moreover, the greater association of delayed source control and defervescence timing among patients who initially presented with a more critical illness might been disclosed in our cohort. In summary, in addition to recognise the advantage of prompt source control on patient prognoses, as consistent with the SSC guideline [3], the present study might expand this therapeutic benefit to include shortening the timing of defervescence for patients with ScR bacteraemia.

This study possesses numerous limitations majorly due to its retrospective and observational design. First, to diminish information bias and improve data accuracy in reviewing medical charts, as previously suggested [28], all data were independently captured by two physicians and these chart abstracters were blind to the aim and hypotheses of our study. To minimise inconsistencies in data capturing, all the recording discrepancies were resolved through direct discussion between the data abstractors. We acknowledge the potential variability in the accuracy of time recordings, particularly in busy clinical settings like the ICU and ED where nurses have numerous tasks to complete on an hourly basis. While efforts were made to record time points accurately, it is possible that some entries may have a margin of error due to the demands of clinical care. Second, to assess the associations of the TtAa or TtSc delay and patient prognoses or defervescence, patients lacking complete data for the exposures (i.e., TtAa and TtSc) and outcomes as well as those lacking complete clinical information (such as image studies and fatality) were excluded in our study design. To reduce the number of excluded patients due to the missing time of appropriate antimicrobial therapy, all etiologic bacteria were prospectively restored and a multicenter design consisting of major hospitals within a city was implemented to reduce the number of cases with incomplete clinical information due to hospital transference. Consequently, a trivial selection bias is expected because only the few proportions of patients (136/5613, 2.4%) were excluded from analyses. Third, despite the presence of missing data during data capture, selection bias resulting from this missing data might be negligible. This is because only three covariates had missing data, and the proportion of missing data for each covariate was ≤ 0.7% Fourth, to accurately incorporate the time of appropriate source control in our analyses, its definition was consistent with previous studies detailing the prognostic advantage of prompt source control [14, 15], and the appropriateness of source control was jointly determined by two physicians in our study design. Fifth, to minimise the interference of confounding factors on study outcomes, a predetermined record form involving all clinical parameters in previous bacteraemia studies and the multivariate regression model were adopted in our analyses. Sixth, we did not assess the adverse impact of broad-spectrum antimicrobial prescription on promoting *Clostridium difficile* infections and antimicrobial resistance as well as the procedure complication linked to emergent processing for source control. Additionally, the cost-saving of rapid defervescence and the shortened length of hospitalisation were not evaluated. Seventh, due to the study period comprising both the Coronavirus disease 2019 (COVID-19) pandemic and the pre-COVID-19 period, the COVID-19 pandemic, which was associated with both TtAa and 30-day mortality, was identified as a confounding factor and was also included for analysis (as shown in Supplemental Tables 2, 3, and 4). Finally, because the study hospitals were localised in southern Taiwan, the absolute value of increasing mortality rates for TtAa or TtSc delay recognised in our cohort may not be externally applied to other communities with varied severity of comorbidities or bacteraemia. Therefore, a prospective study should be conducted to validate these prompt interventions and thereby decrease mortality rates and facilitate rapid defervescence in the future.

## Conclusions

Irrespective of whether bacteraemia necessitated source control or not, our study indicated an association between prompt administration of appropriate antimicrobials and favourable prognosis or rapid defervescence, and these association might be pronounced among critically ill individuals. For individuals experiencing ScR bacteraemia, delayed source control was associated with short-term prognoses and delayed defervescence, and these associations seem to be augmented among those initially exhibiting critical illness. Based on the results of the current study, a prospective study of a large population should be conducted in the future to validate these prompt interventions and thereby decrease mortality rates and facilitate rapid defervescence.

### Supplementary Information

**Additional file 1**.

## Data Availability

Available from the corresponding author on reasonable request.

## Abbreviations

AOR: Adjusted odds ratio
BLI: β-Lactamase inhibitor
BT: Body temperature
COVID-19: Coronavirus disease 2019
CLSI: Clinical and Laboratory Standards Institute
ED: Emergency department
STEDBSI: Southern Taiwan ED BloodStream Infection
ID: Infectious disease
IQR: Interquartile range
OR: Odds ratio
PBS: Pitt bacteraemia score
ScR: Source-control-required
ScU: Source-control-unrequired
SSC: Surviving Sepsis Campaign
TtAa: Time-to-appropriate antibiotic
TtSc: Time-to-source control
