# Supplementary material for: Prompt antimicrobial therapy and source control on survival and defervescence of adults with bacteraemia in the emergency department: the faster, the better
Source: Crit Care. 2024 May 24;28:176. doi: 10.1186/s13054-024-04963-7 (PMC11127347; doi:10.1186/s13054-024-04963-7)
Supplement: Supplementary file 1 — Additional file 1. [file 13054_2024_4963_MOESM1_ESM.pdf]

Supplemental Data

Appendix S1. Appendix describing the source control techniques separated in percutaneous and surgical source control.

Surgical source control: Appendectomy, Gastric surgery, Gastrectomy, Esophagectomy, Gastric or duodenal ulcer suture, Liver surgery, Small intestinal resection, Ileostomy, Colostomy, Hemicolectomy, Sigmoidectomy, Pancreatectomy, Cholecystectomy, Nephrectomy, Bronchus and lung surgery, Intervention on mediastinum, Abscess incision and debridement, Debridement of neck and mediastinum, Fasciotomy, Fasciectomy, Skin-soft tissue intervention, Hysterectomy, Other obstetric surgery, Valvular heart surgery, Cranial surgery, Vascular surgery, Others.

Percutaneous source control: Abdominal drainage, Biliary drainage, Nephrostomy, Ureteral catheterization, Chest tube.

Appendix S2. The types of abscesses identified in varied bacteraemia sources.

Biliary tract: Cholangeal abscess, Gallbladder abscess.

Bone and joint: Extra-articular abscess, Epidural abscess, Paravetebral abscess.

Central-nerve-system: brain abscess.

Ear-nose-throat: deep-neck abscess. Retropharyngeal abscess, Peritonsillar abscess.

Infective endocarditis: Peri-annular abscess, Valve ring abscess.

Intraabdominal: Intraabdominal abscess, Peritoneal abscess.

Liver abscess: Intra-hepatic abscess

Low respiratory tract: Lung abscess, Empyema.

Pelvic inflammatory disease: Pelvic abscess, Tubo-ovarian abscess.

Skin and soft-tissue: Pyomyositis, Intramuscular abscess, Subcutaneous abscess,

Urinary tract: Renal abscess, Pararenal abscess, Prostate abscess.

Appendix S3.

The majorities of Gram-positive aerobes were *Streptococcus* species (753 isolates, 12.0%), *Staphylococcus aureus* (650, 10.4%), and *Enterococcus* species (165, 2.6%). The leading five Gram-negative aerobes were *Escherichia coli* (2019 isolates, 32.3%), *Klebsiella pneumoniae* (1250, 20.0%), *Pseudomonas* species (177, 2.8%), *Proteus* species (129, 2.1%), and *Enterobacter* species (117, 1.9%). Anaerobes accounted for only 4.1% (255 isolates) of the total pathogens. Methicillin-resistant *S. aureus* and ampicillin-susceptible enterococci accounted for 38.6% (251 isolates) of *S. aureus* and 84.8% (140) of enterococci, respectively. Of the streptococci, 91.1% (686 isolates) were susceptible to penicillin. Overall, imipenem, piperacillin/tazobactam, cefepime, ertapenem, ceftazidime, levofloxacin, cefotaxime, moxifloxacin, ampicillin/sulbactam, cefuroxime, or cefazolin was active against 100%, 94.6%, 93.0%, 91.7%, 86.6%, 86.2%, 82.1%, 81.3%, 79.1%, 74.2%, or 57.6%, respectively, of Gram-negative aerobes. Metronidazole, piperacillin/tazobactam, moxifloxacin, or ampicillin/sulbactam was, respectively, active against 98.6%, 98.0%, 92.5%, or 83.0% of the total anaerobes, respectively.

Appendix S4.

The majorities of the initial antimicrobials administered were third-generation cephalosporins (3239 patients, 50.2%), followed by second-generation cephalosporins (633, 9.8%), fourth-generation cephalosporins (516, 8.0%), aminopenicillin/β-lactamase inhibitor (BLI) (505, 7.8%), ureidopenicillin/BLI (420, 6.5%), glycopeptides (378, 5.9%), fluoroquinolones (287, 4.4%), carbapenems (208, 3.2%), ureidopenicillins (108, 1.7%), metronidazole (71, 1.1%) , aminopenicillin (35, 0.5%), first-generation cephalosporins (31, 0.5%), and others (22, 0.3%).

Supplemental Figure 1. Flowchart of patient selections.

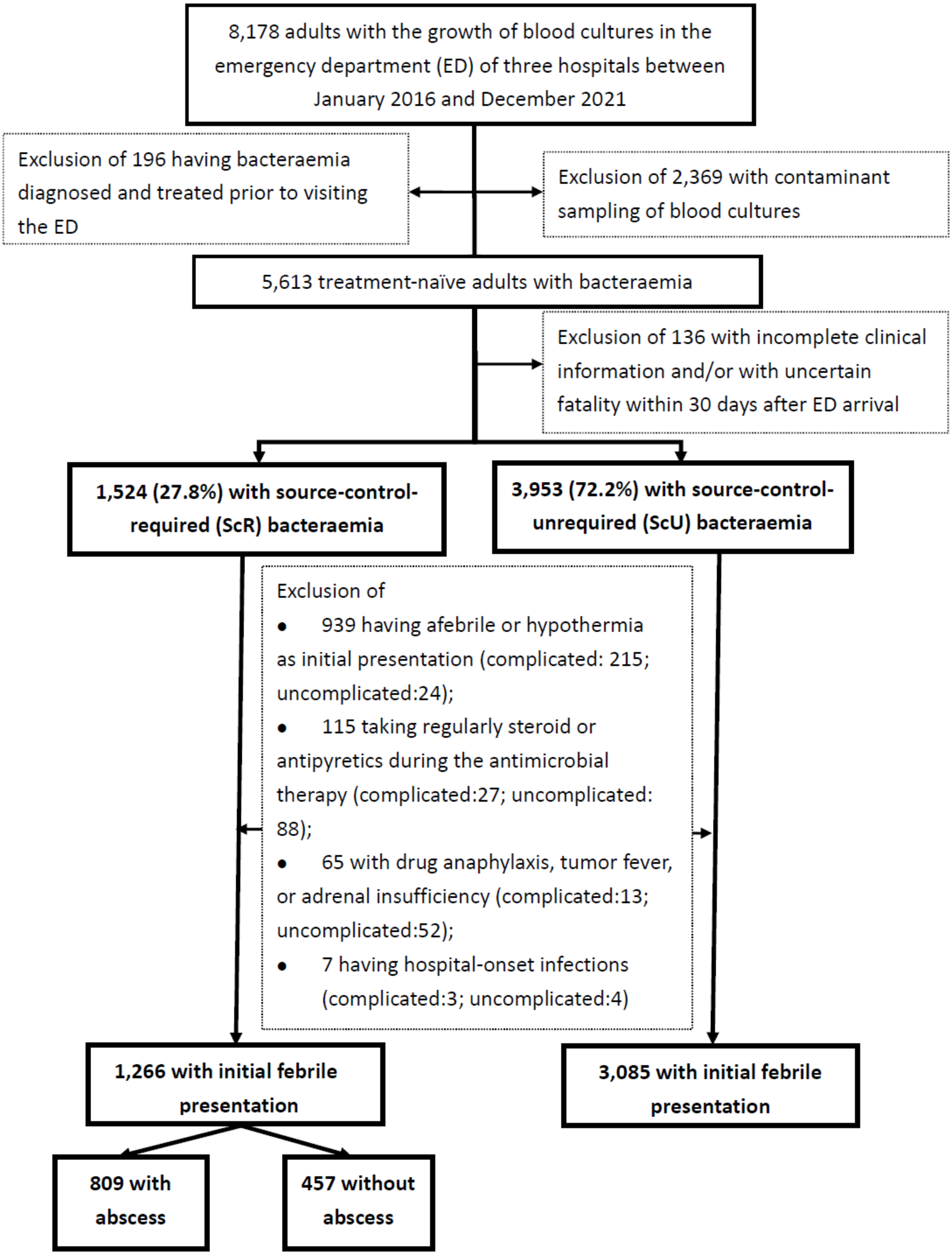

Supplemental Figure 2. The missing proportion of variables of interest in the study.

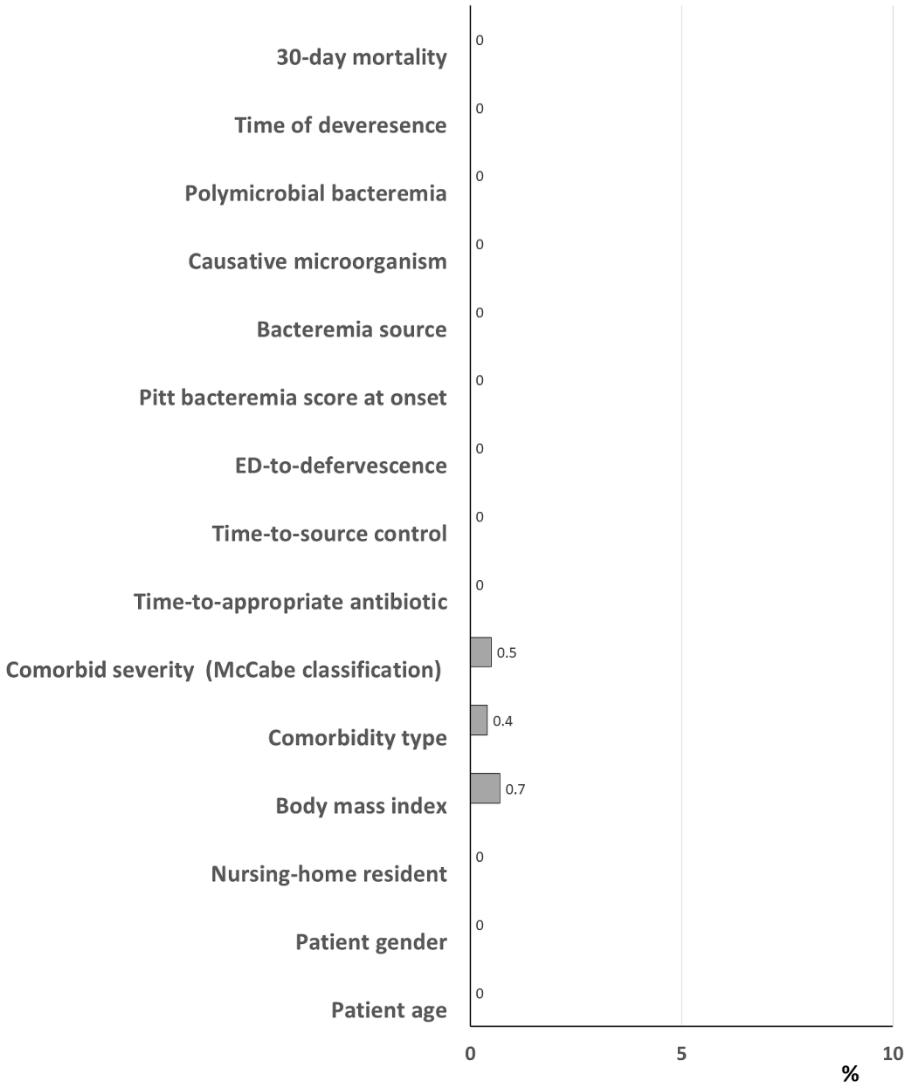

Supplemental Figure 3. A positive trend of 30-day crude mortality rates in varied periods of the time-to-appropriate antibiotic for source-control-unrequired bacteraemia (Fig 3A) and source-control-required bacteraemia (Fig 3B) bacteraemia and that in varied periods of the time-to-source control for complicated bacteraemia (Fig 3C)*.

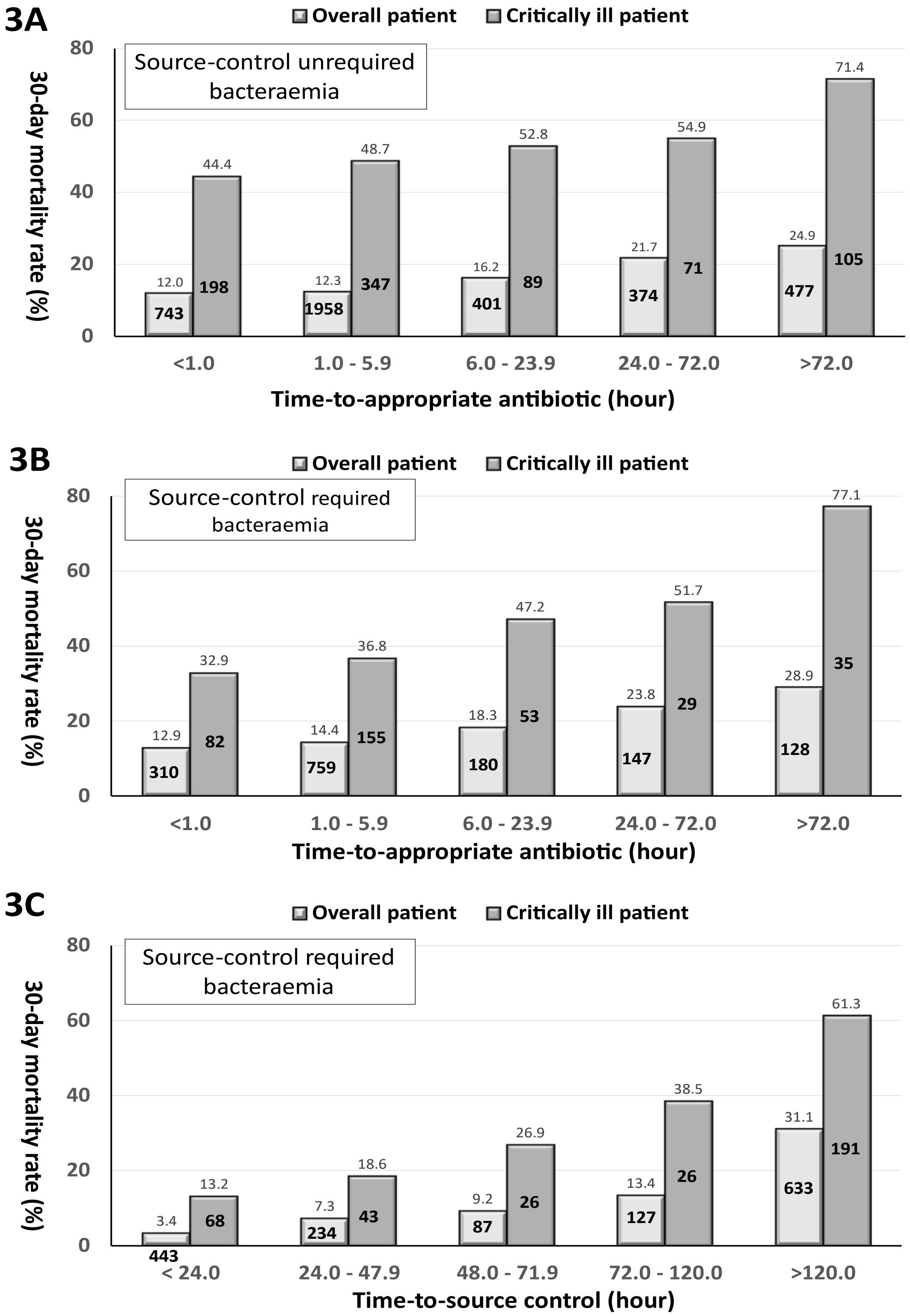

*Numbers in the box indicated the patient numbers.

Supplemental Figure 4. A positive trend of the patient proportion of delayed defervescence in varied periods of the time-to-appropriate antibiotic for source-control-unrequired bacteraemia (Fig 4A) and source-control-required bacteraemia (Fig 4B) bacteraemia and that in varied periods of the time-to-source control for source-control required bacteraemia (Fig 4C)*.

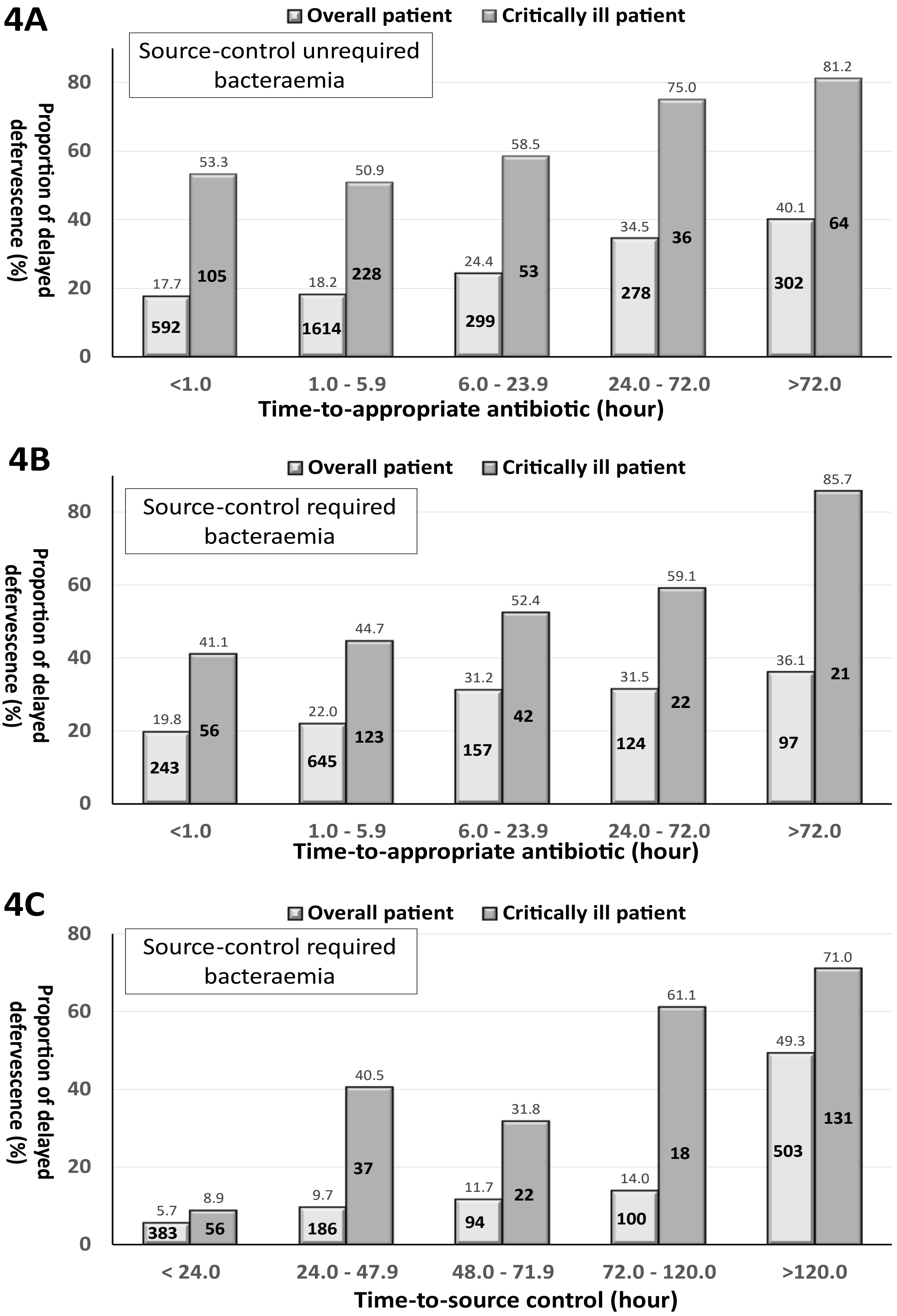

*Only patients having initial febrile presentation were calculated and numbers in the box indicated the patient numbers.

Supplemental Table 1. STROBE Statement Checklist.

	Item No	Recommendation	Page No
**Title and abstract**	1	(a) Indicate the study's design with a commonly used term in the title or the abstract	3
		(b) Provide in the abstract an informative and balanced summary of what was done and what was found	23
**Introduction**			
Background/rationale	2	Explain the scientific background and rationale for the investigation being reported	4
Objectives	3	State specific objectives, including any prespecified hypotheses	4-5
**Methods**			
Study design	4	Present key elements of study design early in the paper	7
Setting	5	Describe the setting, locations, and relevant dates, including periods of recruitment, exposure, follow-up, and data collection	7-8
Participants	6	(a) Give the eligibility criteria, and the sources and methods of selection of participants. Describe methods of follow-up	7
		(b) For matched studies, give matching criteria and number of exposed and unexposed	NA
Variables	7	Clearly define all outcomes, exposures, predictors, potential confounders, and effect modifiers. Give diagnostic criteria, if applicable	9-10
Data sources/ measurement	8*	For each variable of interest, give sources of data and details of methods of assessment (measurement). Describe comparability of assessment methods if there is more than one group	7-8
Bias	9	Describe any efforts to address potential sources of bias	8
Study size	10	Explain how the study size was arrived at	
Quantitative variables	11	Explain how quantitative variables were handled in the analyses. If applicable, describe which groupings were chosen and why	10-11
Statistical methods	12	(a) Describe all statistical methods, including those used to control for confounding	10-11
		(b) Describe any methods used to examine subgroups and interactions	7, 11

		(c) Explain how missing data were addressed	8
		(d) If applicable, explain how loss to follow-up was addressed	7
		(e) Describe any sensitivity analyses	NA
**Results**			
Participants	13*	(a) Report numbers of individuals at each stage of study—eg numbers potentially eligible, examined for eligibility, confirmed eligible, included in the study, completing follow-up, and analysed	Figure 1
		(b) Give reasons for non-participation at each stage	Figure 1
		(c) Consider use of a flow diagram	Figure 1
Descriptive data	14*	(a) Give characteristics of study participants (eg demographic, clinical, social) and information on exposures and potential confounders	12-13
		(b) Indicate number of participants with missing data for each variable of interest	Suppl. Figure 1
		(c) Summarise follow-up time (eg, average and total amount)	16
Outcome data	15*	Report numbers of outcome events or summary measures over time	13-15
Main results	16	(a) Give unadjusted estimates and, if applicable, confounder-adjusted estimates and their precision (eg, 95% confidence interval). Make clear which confounders were adjusted for and why they were included	15-16
		(b) Report category boundaries when continuous variables were categorised	16
		(c) If relevant, consider translating estimates of relative risk into absolute risk for a meaningful time period	NA
Other analyses	17	Report other analyses done—eg analyses of subgroups and interactions, and sensitivity analyses	15-16
**Discussion**			
Key results	18	Summarise key results with reference to study objectives	17
Limitations	19	Discuss limitations of the study, taking into account sources of potential bias or imprecision. Discuss both direction and magnitude of any potential bias	19
Interpretation	20	Give a cautious overall interpretation of results considering objectives, limitations, multiplicity of analyses, results from similar studies, and other relevant evidence	19

Generalisability	21	Discuss the generalisability (external validity) of the study results	19
**Other information**			
Funding	22	Give the source of funding and the role of the funders for the present study and, if applicable, for the original study on which the present article is based	21

Supplemental Table 2. Risk factors of 30-day crude mortality in patients with source-control-unrequired (ScU) bacteraemia*.

Variables	Patient number (%)		Univariate analysis		Multivariate analysis	
	Death, n=595	Survival, n=3358	OR (95% CI)	P value	Adjusted OR (95% CI)	*P* value
The elderly, ≥ 65 years	382 (64.2)	2012 (59.9)	1.20 (0.00 – 1.44)	0.049	1.24 (0.97 – 1.58)	0.09
Gender, male	352 (59.2)	1639 (48.8)	1.52 (1.27 – 1.81)	<0.001	NS	NS
Body mass index < 18.5 kg/m^2	146 (24.5)	404 (12.0)	2.38 (1.92 – 2.94)	<0.001	NS	NS
**Nursing-home resident**	**82 (13.8)**	**141 (4.2)**	**3.65 (2.74 – 4.86)**	**<0.001**	**1.91 (1.28 – 2.84)**	**0.001**
The COVID-19 pandemic	132 (22.2)	903 (26.9)	0.78 (0.63 – 0.95)	0.02	NS	NS
**Pitt bacteraemia score ≥4 at onset**	**418 (70.3)**	**392 (11.7)**	**17.87 (14.56 – 21.93)**	**<0.001**	**9.38 (7.35 – 11.95)**	**<0.001**
Polymicrobial bacteraemia	85 (14.3)	205 (6.1)	2.56 (1.96 – 3.36)	<0.001	NS	NS
Bacteraemia source						
**Low respiratory tract**	**402 (67.6)**	**419 (12.5)**	**14.61 (11.96 – 17.84)**	**<0.001**	**3.67 (2.72 – 4.95)**	**<0.001**
**Urinary tract**	**49 (8.2)**	**1479 (44.0)**	**0.11 (0.08 – 0.15)**	**<0.001**	**0.22 (0.08 – 0.57)**	**0.002**
**Primary bacteraemia**	**29 (4.9)**	**290 (8.6)**	**0.54 (0.37 – 0.80)**	**0.002**	**0.51 (0.32 – 0.83)**	**0.007**
Skin and soft-tissue	6 (1.0)	252 (7.5)	0.13 (0.06 – 0.28)	<0.001	NS	NS
Biliary tract	5 (0.8)	188 (5.6)	0.14 (0.06 – 0.35)	<0.001	NS	NS
Etiologic pathogen						
Klebsiella pneumoniae	166 (27.9)	596 (17.7)	1.79 (1.47 – 2.19)	<0.001	1.27 (0.97 – 1.68)	0.09
Escherichia coli	131 (22.0)	1439 (42.9)	0.38/ (0.31 – 0.46)	<0.001	NS	NS
Staphylococcus aureus	91 (15.3)	329 (9.8)	1.66 (1.29 – 2.14)	<0.001	NS	NS
Pseudomonas species	49 (8.2)	100 (3.0)	2.92 (2.05 – 4.16)	<0.001	1.57 (0.96 – 2.56)	0.07
**Fatal comorbidity (McCabe classification)**	**301 (50.6)**	**770 (22.9)**	**3.44 (2.88 – 4.12)**	**<0.001**	**2.11 (1.52 – 2.91)**	**<0.001**
Comorbidity						
Cardiovascular disease	283 (47.6)	1871 (55.7)	0.72 (0.61 – 0.86)	<0.001	NS	NS
**Malignancy**	**201 (33.8)**	**467 (13.9)**	**3.16 (2.60 – 3.84)**	**<0.001**	**2.14 (1.50 – 3.05)**	**<0.001**

Neurological disease	191 (32.1)	785 (23.4)	1.55 (1.28 – 1.87)	<0.001	NS	NS
**Liver cirrhosis**	**112 (18.8**	**377 (11.2)**	**1.83 (1.45 - 2.31)**	**<0.001**	**3.67 (1.54 – 8.75)**	**0.003**

CI = confidence interval; NS = not significant (after processing the backward multivariate regression); OR = odds ratio.

* Boldface indicates independent predictors of 30-day mortality, which are statistical significance (*P* < 0.05) under the multivariate regression model.

Supplemental Table 3. Risk factors of 30-day crude mortality in patients with source-control-required bacteraemia*.

Variables	Patient number (%)		Univariate analysis		Multivariate analysis	
	Death, n=254	Survival, n=1270	OR (95% CI)	P value	Adjusted OR (95% CI)	*P* value
**The elderly, ≥ 65 years**	**173 (68.1)**	**722 (56.9)**	**1.62 (1.22 – 2.16)**	**0.001**	**1.48 (1.04 – 2.09)**	**0.03**
Nursing- home resident	16 (6.3)	35 (2.8	2.37 (1.29 – 4.36)	0.004	1.92 (0.94 – 3.93)	0.07
Body mass index < 18.5 kg/m^2	50 (19.7)	149 (11.7)	1.84 (1.30 – 2.63)	0.001	NS	NS
The COVID-19 pandemic	81 (31.9)	492 (38.7)	0.74 (0.56 – 0.99)	0.04	NS	NS
**Polymicrobial bacteraemia**	**54 (21.3)**	**137 (10.8)**	**2.23 (1.58 – 3.17)**	**<0.001**	**1.65 (1.07 – 2.54)**	**0.02**
**Pitt bacteraemia score ≥4 at onset**	**151 (59.4)**	**203 (16.0)**	**7.71 (5.75 – 10.32)**	**<0.001**	**7.58 (5.41 – 10.61)**	**<0.001**
Bacteraemia source						
**Skin and soft-tissue**	**77 (30.3)**	**285 (22.4)**	**1.50 (1.12 – 2.03)**	**0.007**	**1.68 (1.11 – 2.53)**	**0.01**
**Intra-abdomen**	**65 (25.6)**	**175 (13.8)**	**2.15 (1.56 – 2.98)**	**<0.001**	**2.08 (1.37 – 3.16)**	**0.001**
**Low respiratory tract**	**45 (17.7)**	**80 (6.3)**	**3.20 (2.16 – 4.75)**	**<0.001**	**1.95 (1.19 – 3.21)**	**0.008**
Biliary tract	33 (13.0)	321 (25.3)	0.44 (0.30 – 0.65)	<0.001	NS	NS
**Bone and joint**	**16 (6.3)**	**146 (11.5)**	**0.52 (0.30 – 0.88)**	**0.01**	**0.42 (0.22 – 0.81)**	**0.009**
**Liver abscess**	**12 (4.7)**	**196 (15.4)**	**0.27 (0.15 – 0.50)**	**<0.001**	**0.40 (0.20 - 0.80)**	**0.01**
Etiologic pathogen						
Klebsiella pneumoniae	64 (25.2)	423 (33.3)	0.67 (0.50 – 0.92)	0.01	0.68 (0.45 – 1.02)	0.06
Escherichia coli	**60 (23.6)**	**388 (30.3)**	**0.70 (0.51 – 0.96)**	**0.03**	**0.59 (0.39 – 0.89)**	**0.01**
Staphylococcus aureus	**51 (20.1)**	**178 (14.0)**	**1.54 (1.09 – 2.18)**	**0.01**	**1.68 (1.03 – 2.74)**	**0.04**
Anaerobes	34 (13.4)	90 (7.1)	2.03 (1.33 – 3.08)	0.001	NS	NS
Pseudomonas species	11 (4.3)	17 (1.3)	3.34 (1.54 – 7.21)	0.003	NS	NS
**Fatal comorbidity (McCabe classification)**	**112 (44.1)**	**222 (17.5)**	**3.72 (2.80 – 4.96)**	**<0.001**	**1.85 (1.17 – 2.93)**	**<0.001**
Comorbidity						

**Malignancy**	**72 (28.3)**	**147 (11.6)**	**3.02 (2.19 - 4.17)**	**<0.001**	**1.68 (1.11 – 2.53)**	**0.01**
**Chronic kidney disease**	**77 (30.3)**	**253 (19.9)**	**1.75 (1.29 – 2.36)**	**<0.0001**	**1.54 (1.06 – 2.24)**	**0.02**
Neurological disease	63 (24.8)	245 (19.3)	1.38 (1.01 – 1.89)	0.046	NS	NS
Liver cirrhosis	45 (17.7)	113 (8.9)	2.21 (1.51 – 3.21)	<0.001	NS	NS

CI = confidence interval; NS = not significant (after processing the backward multivariate regression); OR = odds ratio.

* Boldface indicates independent predictors of 30-day mortality, which are statistical significance ($P < 0.05$) under the multivariate regression model.

Supplemental Table 4. Predictors of 30-day crude mortality in critically ill patients*.

Variables	Patient number (%)		Univariate analysis		Multivariate analysis	
	Death	Survival	OR (95% CI)	P value	Adjusted OR (95% CI)	*P* value
Source-control-unrequired bacteraemia, n = 810	n = 418	n = 392				
Gender, male	250 (59.8)	301 (51.3)	1.41 (1.07 1.87)	0.002	NS	NS
Etiologic pathogen						
Klebsiella pneumoniae	123 (29.4)	76 (19.4)	1.73 (1.25 – 2.40)	0.001	NS	NS
Escherichia coli	85 (20.3)	148 (37.8)	0.42 (0.31 – 0.58)	<0.001	NS	NS
Streptococcus species	80 (19.1)	51 (13.0)	1.58 (1.08 – 2.32)	0.02	NS	NS
Pseudomonas species	35 (8.4)	15 (3.8)	2.30 (1.23 – 4.28)	0.007	NS	NS
***Enterococcus* species**	**5 (1.2)**	**17 (4.3)**	**0.27 (0.10 – 0.73)**	**0.006**	**0.23 (0.08 – 0.69)**	**0.009**
Bacteraemia source						
**Low respiratory tract**	**325 (77.8)**	**141 (36.0)**	**6.22 (4.57 – 8.48)**	**<0.001**	**3.05 (2.06 – 4.52)**	**<0.001**
**Urinary tract**	**24 (5.7)**	**148 (37.8)**	**0.10 (0.06 – 0.16)**	**<0.001**	**0.21 (0.12 – 0.37)**	**<0.001**
**Skin and soft-tissue**	**2 (0.5)**	**14 (3.6)**	**0.143 (0.03 – 0.58)**	**0.02**	**0.20 (0.20 – 0.90)**	**0.04**
Biliary tract	0 (0)	11 (2.8)	–	0.001	NS	NS
Fatal comorbidity (McCabe classification)	182 (43.5)	105 (26.8)	2.11 (1.57 – 2.83)	<0.001	1.53 (0.99 – 2.35)	0.06
**Comorbid malignancy**	**125 (29.9)**	**53 (13.5)**	**2.73 (1.91 – 3.90)**	**<0.001**	**1.72 (1.04 – 2.84)**	**0.03**
Source-control-required bacteraemia, n = 354	n = 151	n = 203				
The COVID-19 pandemic	42 (27.8)	80 (39.4)	0.59 (0.38 – 0.93)	0.02	NS	NS
**Etiologic pathogen of *Klebsiella pneumoniae***	**37 (24.5)**	**91 (44.8)**	**0.40 (0.25 – 0.63)**	**<0.001**	**0.45 (0.27 – 0.75)**	**0.002**
Bacteraemia source						

Intra-abdominal	44 (29.1)	34 (16.7)	2.04 (1.23 3.40)	0.005	1.63 (0.93 – 2.85)	0.09
**Liver abscess**	**9 (6.0)**	**44 (21.7)**	**0.23 (0.11 0.49)**	**<0.001**	**0.33 (0.15 – 0.76)**	**0.009**
**Bone and joint**	**6 (4.0)**	**21 (10.3)**	**0.36 (0.14 0.91)**	**0.03**	**0.29 (0.11 – 0.77)**	**0.01**
Fatal comorbidity (McCabe classification)	58 (38.4)	39 (19.2)	2.62 (1.62 – 4.23)	<0.001	NS	NS
Comorbidity						
Diabetes mellitus	61 (40.4)	107 (52.7)	0.61 (0.40 – 0.93)	0.02	NS	NS
**Malignancy**	**36 (23.8)**	**20 (9.9)**	**2.86 (1.58 – 5.19)**	**<0.001**	**3.03 (1.61 – 5.67)**	**0.001**
**Liver cirrhosis**	**24 (15.9)**	**15 (7.4)**	**2.37 (1.20 – 4.69)**	**0.01**	**2.97 (1.42 – 6.21)**	**0.004**

CI = confidence interval; NS = not significant (after processing the backward multivariate regression); OR = odds ratio. * Boldface indicates independent predictors of 30-day mortality, which are statistical significance ($P < 0.05$) under the multivariate regression model.

Supplemental Table 5 Predictors of delayed defervescence (ED-to-defervescence > 8 days) in 3085 febrile adults with source-control-unrequired bacteraemia*.

Clinical variables	Patient number with delayed defervescence (%)		Univariate analysis		Multivariate analysis	
	Yes, n=689	No, n=2396	OR (95% CI)	*P* value	AOR (95% CI)	*P* value
Gender, male	372 (54.0)	1112 (46.4)	1.36 (1.14 – 1.61)	<0.001	NS	NS
Nursing-home resident	54 (7.8)	85 (3.5)	2.31 (1.63 – 3.29)	<0.001	NS	NS
Body mass index < 18.5 kg/m^2	113 (16.4)	256 (10.7)	1.64 (1.29 – 2.08)	<0.001	NS	NS
**Polymicrobial bacteraemia**	**67 (9.7)**	**128 (5.3)**	**1.91 (1.40 - 2.60)**	**<0.001**	**1.56 (1.07 – 2.29)**	**0.02**
**Pitt bacteraemia score ≥4 at onset**	**282 (40.9)**	**204 (8.5)**	**7.45 (6.04 – 9.18)**	**<0.001**	**4.40 (3.44 – 5.62)**	**<0.001**
Bacteraemia source						
**Low respiratory tract**	**292 (42.4)**	**166 (6.9)**	**9.88 (7.94 - 12.29)**	**<0.001**	**6.81 (5.31 – 8.73)**	**<0.001**
Urinary tract	148 (21.5)	1172 (48.9)	0.29 (0.23 – 0.35)	<0.001	NS	NS
**Infective endocarditis**	**52 (7.5)**	**34 (1.4)**	**5.67 (3.65 - 8.82)**	**<0.001**	**8.35 (5.306 – 13.76)**	**<0.001**
Skin and soft-tissue	24 (3.5)	194 (8.1)	0.41 (0.27 – 0.63)	<0.001	NS	NS
**Bone and joint**	**20 (2.9)**	**29 (1.2)**	**2.44 (1.37 – 4.34)**	**0.002**	**4.18 (2.20 – 7.93)**	**<0.001**
Central nerve system	15 (2.2)	5 (0.2)	10.64 (3.85 – 29.39)	<0.001	NS	NS
Biliary tract	14 (2.0)	148 (6.2)	0.32 (0.18 – 0.55)	<0.001	NS	NS
Etiologic pathogen						
Escherichia coli	198 (28.7)	1128 (47.1)	0.45 (0.38 – 0.55)	<0.001	NS	NS
Staphylococcus aureus	**117 (17.0)**	**166 (6.9)**	**2.75 (2.13 – 3.54)**	**<0.001**	**1.75 (1.27 – 2.42)**	**0.001**
***Pseudomonas* species**	**49 (7.1)**	**62 (2.6)**	**2.88 (1.96 – 4.24)**	**<0.001**	**1.96 (1.22 – 3.17)**	**0.006**
Anaerobes	25 (3.6)	42 (1.8)	2.11 (1.28 – 3.49)	0.003	NS	NS
**Fatal comorbidity (McCabe classification)**	**258 (37.4)**	**498 (20.8)**	**2.28 (1.90 – 2.74)**	**<0.001**	**2.12 (1.68 – 2.68)**	**<0.001**
Comorbidity						

Neurological disease	195 (28.3)	508 (21.2)	1.47 (1.21 - 1.78)	<0.001	NS	NS
Chronic kidney disease	160 (23.2)	449 (18.7)	1.31 (1.07 - 1.61)	0.009	1.25 (0.98 – 1.59)	0.08
Malignancy	157 (22.8)	313 (13.1)	1.96 (1.59 – 2.43)	<0.001	NS	NS
Liver cirrhosis	95 (13.8)	265 (11.1)	1.29 91.00 – 1.65)	0.049	1.32 (0.97 – 1.80)	0.08
Chronic obstructive pulmonary disease	41 (6.0)	99 (4.1)	1.49 (1.01 – 2.13)	0.04	NS	NS

AOR = adjusted odds ratio; CI = confidence interval; NS = not significant (by backward multivariate regression); OR = odds ratio. * Boldface indicates independent predictors of delayed defervescence, which are statistical significance ($P < 0.05$) under the multivariate regression model.

Supplemental Table 6. Predictors of delayed defervescence (ED-to-defervescence > 20 days) in 1271 febrile adults with source-control-required bacteraemia*.

Clinical variables	Patient number with delayed defervescence (%)		Univariate analysis		Multivariate analysis	
	Yes, n=313	No, n=953	OR (95% CI)	*P* value	AOR (95% CI)	*P* value
**Pitt bacteraemia score ≥4 at onset**	**131 (41.9)**	**133 (14.0)**	**4.44 (3.32 – 5.93)**	**<0.001**	**4.45 (3.21 – 6.17)**	**<0.001**
Bacteraemia source						
**Skin and soft-tissue**	**100 (31.9)**	**186 (19.5)**	**1.94 (1.45 – 2.58)**	**<0.001**	**1.51 (1.04 – 2.19)**	**0.03**
Intra-abdominal	80 (19.2)	136 (14.3)	1.43 (1.02 – 1.99)	0.04	1.50 (0.99 – 2.28)	0.06
**Bone and joint**	**53 (16.9)**	**72 (7.6)**	**2.49 (1.70 - 3.65)**	**<0.001**	**2.47 (1.58 – 3.87)**	**<0.001**
**Low respiratory tract**	**53 (16.9)**	**45 (4.7)**	**4.11 (2.70 – 6.26)**	**<0.001**	**2.94 (1.81 – 4.78)**	**<0.001**
**Biliary tract**	**27 (8.6)**	**276 (29.0)**	**0.23 (0.15 - 0.35)**	**<0.001**	**0.40 (0.25 – 0.65)**	**<0.001**
**Liver abscess**	**27 (8.6)**	**167 (17.5)**	**0.44 (0.29 - 0.68)**	**<0.001**	**0.49 (0.30 – 0.82)**	**0.006**
**Mycotic aneurysm**	**14 (4.5)**	**5 (0.5)**	**8.88 (3.17 – 24.85)**	**<0.001**	**10.16 (3.28 – 31.46)**	**<0.001**
Infective endocarditis	10 (3.2)	10 (1.0)	3.11 (1.28 – 7.55)	0.02	NS	NS
Etiologic pathogen						
Staphylococcus aureus	66 (21.1)	108 (11.3)	2.09 (1.49 - 2.93)	<0.001	NS	NS
Escherichia coli	64 (20.4)	307 (32.2)	0.54 (0.40 – 0.74)	<0.001	NS	NS
Streptococcus species	43 (13.7)	81 (8.5)	1.71 (1.16 – 2.54)	0.007	NS	NS
Salmonella species	8 (2.6)	4 (0.4)	6.22 (1.86 - 20.81)	0.001	NS	NS
Fatal comorbidity (McCabe classification)	95 (30.4)	157 (16.5)	2.21 (1.64 – 2.97)	<0.001	NS	NS
Comorbidity						
**Chronic kidney disease**	**97 (31.0)**	**159 (16.7)**	**2.24 (1.67 – 3.01)**	**<0.001**	**1.95 (1.40 – 2.73)**	**<0.001**
Neurological disease	73 (23.3)	172 (18.0)	1.38 (1.01 – 1.88)	0.04	NS	NS
**Malignancy**	**61 (19.5)**	**105 (11.0)**	**1.96 (1.38 – 2.76)**	**<0.001**	**2.79 (1.86 – 4.18)**	**<0.001**

**Liver cirrhosis**	**50 (16.0)**	**81 (8.5)**	**2.058 (1.40 - 2.99)**	**<0.001**	**2.20 (1.44 - 3.39)**	**<0.001**

AOR = adjusted odds ratio; CI = confidence interval; NS = not significant (by backward multivariate regression); OR = odds ratio. *Boldface indicates independent predictors of delayed defervescence, which are statistical significance ($P < 0.05$) under the multivariate regression model.

Supplemental Table 7. Predictors of delayed defervescence in febrile patients initial presented with the critical illness, categorised into the source-control-unrequired bacteraemia and source-control-required bacteraemia*.

Variables	Patient No. with delayed defervescence (%)		Univariate analysis		Multivariate analysis	
	Yes	No	OR (95% CI)	P value	Adjusted OR (95% CI)	*P* value
Source-control-unrequired bacteraemia, n = 486	n = 282	n = 204				
Etiologic pathogen						
Klebsiella pneumoniae	78 (27.7)	39 (19.1)	1.62 (1.05 – 2.50)	0.03	NS	NS
Escherichia coli	64 (22.7)	93 (45.6)	0.35 (0.24 – 0.52)	<0.001	NS	NS
Streptococcus species	47 (16.7)	23 (11.3)	1.57 (0.92 – 2.69)	0.095	NS	NS
Pseudomonas species	25 (8.9)	7 (3.4)	2.74 (1.16 – 6.46)	0.02	NS	NS
**Staphylococcus aureus**	**42 (14.9)**	**11 (5.4)**	**3.07 (1.54 – 6.12)**	**0.001**	**2.37 (1.12 – 5.01)**	**0.02**
Proteus species	4 (1.4)	11 (5.4)	0.25 (0.08 – 0.81)	0.01	NS	NS
Anaerobes	16 (5.7)	3 (1.5)	4.03 (1.16 – 14.02)	0.02	NS	NS
Bacteraemia source						
**Low respiratory tract**	**184 (65.2)**	**49 (24.0)**	**5.94 (2.97 – 8.90)**	**<0.001**	**4.03 (2.43 – 6.67)**	**<0.001**
**Urinary tract**	**40 (14.2)**	**96 (47.1)**	**0.19 (0.12 – 0.29)**	**<0.001**	**0.47(0.27 - 0.81)**	**0.02**
Central nerve system	6 (2.1)	0 (0)		0.04	NS	NS
Biliary tract	1 (0.4)	4 (4.4)	0.08 (0.01 - 0.61)	0.002	0.13 (0.02 – 1.06)	0.06
Fatal comorbidity (McCabe classification)	98 (34.8)	54 (26.5)	1.48 (1.00 – 2.20)	0.05	NS	NS
Comorbid malignancy	60 (21.3)	25 (12.3)	1.94 (1.17 – 3.21)	0.01	NS	NS
Source-control-required bacteraemia, n = 264	n = 131	n = 133				

**Etiologic pathogen of streptococci**	**18 (13.7)**	**8 (6.0)**	**2.49 (1.04 – 5.95)**	**0.04**	**2.53 (1.02 – 6.27)**	**0.046**
Bacteraemia source						
**Low respiratory tract**	**30 (22.9)**	**13 (9.8)**	**2.74 (1.36 - 5.54)**	**0.004**	**2.60 (1.25 – 5.40)**	**0.01**
**Liver abscess**	**18 (13.7)**	**32 (24.1)**	**0.50 (0.27 – 0.95)**	**0.03**	**0.49 (0.25 – 0.97)**	**0.04**
Bone and joint	13 (9.9)	5 (3.8)	2.82 (0.98 – 8.15)	0.047	NS	NS
Biliary tract	12 (9.2)	24 (18.0)	0.46 (0.22 – 0.96)	0.04	0.47 (0.21 – 1.02)	0.06
Fatal comorbidity (McCabe classification)	38 (28.0)	26 (19.5)	1.68 (0.95 – 2.98)	0.07	NS	NS
Comorbidity						
Malignancy	24 (18.3)	14 (10.5)	1.91 (0.94 – 3.87)	0.07	NS	NS
**Liver cirrhosis**	**21 (16.0)**	**7 (5.3)**	**3.44 (1.41 – 8.39)**	**0.005**	**3.60 (1.44 – 8.98)**	**0.006**

CI = confidence interval; NS = not significant (after processing the backward multivariate regression); OR = odds ratio. * Boldface indicates independent predictors of delayed defervescence, which are statistical significance ($P < 0.05$) under the multivariate regression model.

Supplemental Table 8. Predictors of delayed defervescence (ED-to-defervescence > 20 days) in febrile adults with source-control-required bacteraemia, categorised into the abscess and non-abscess groups*.

Clinical variables	Patient number with delayed defervescence (%)		Univariate analysis		Multivariate analysis	
	Yes	No	OR (95% CI)	*P* value	Adjusted OR (95% CI)	*P* value
Abscess group, n = 809	n = 250	n = 559				
**Pitt bacteraemia score ≥4 at onset**	**107 (42.8)**	**89 (15.9)**	**3.95 (2.82 – 5.54)**	**<0.001**	**4.24 (2.91 – 6.17)**	**<0.001**
Bacteraemia source						
**Low respiratory tract**	**50 (20.0)**	**44 (7.9)**	**2.93 (1.89 – 4.53)**	**<0.001**	**2.13 (1.32 – 3.45)**	**0.002**
**Bone and joint**	**41 (16.4)**	**58 (10.4)**	**1.70 (1.10 – 2.61)**	**0.02**	**2.07 (1.32 – 3.26)**	**0.002**
**Liver abscess**	**27 (10.8)**	**167 (29.9)**	**0.28 (0.18 – 0.44)**	**<0.001**	**0.33 (0.20 – 0.54)**	**<0.001**
Infective endocarditis	10 (4.0)	10 (1.8)	2.29 (0.94 – 5.57)	0.06	NS	NS
Etiologic pathogen						
Klebsiella pneumoniae	74 (29.6)	222 (39.7)	0.64 (0.46 – 0.88)	0.006	NS	NS
Staphylococcus aureus	63 (25.2)	96 (17.2)	1.63 (1.13 – 2.33)	0.008	NS	NS
Salmonella species	7 (2.8)	4 (0.7)	4.00 (1.16 – 13.78)	0.04	3.77 (0.97 – 14.62)	0.06
Fatal comorbidity (McCabe classification)	74 (29.6)	88 (15.7)	2.25 (1.58 – 3.21)	<0.001	NS	NS
Comorbidity						
**Chronic kidney disease**	**80 (44.2)**	**101 (18.1)**	**2.13 (1.52 – 3.00)**	**<0.001**	**2.46 (1.48 – 4.08)**	**0.001**
**Liver cirrhosis**	**44 (17.6)**	**51 (9.1)**	**2.13 (1.38 – 3.29)**	**0.001**	**2.06 (1.28 – 3.32)**	**0.003**
Malignancy	41 (16.4)	48 (8.6)	2.09 (1.34 – 3.27)	0.001	NS	NS
Non-abscess group, n = 457	n = 63	n = 394				

**The elderly, ≥ 65 years**	**51 (81.0)**	**264 (67.0)**	**2.09 (1.08 – 4.06)**	**0.03**	**2.25 (1.08 – 4.69)**	**0.03**
**Pitt bacteraemia score ≥4 at onset**	**24 (38.1)**	**44 (11.2)**	**4.90 (2.69 – 8.90)**	**<0.001**	**4.65 (2.40 – 9.00)**	**<0.001**
Bacteraemia source						
Urinary tract	32 (50.8)	113 (28.7)	2.57 (1.50 – 4.41)	0.001	NS	NS
**Biliary tract**	**21 (33.3)**	**261 (66.2)**	**0.26 (0.15 – 0.45)**	**<0.001**	**0.32 (0.17 – 0.59)**	**<0.001**
**Low respiratory tract**	**3 (4.8)**	**1 (0.3)**	**9.65 (2.01 – 192.00)**	**0.009**	**33.1 (2.84 – 384.59)**	**0.005**
Fatal comorbidity (McCabe classification)	21 (33.3)	69 (17.5)	2.36 (1.31 – 4.23)	0.003	NS	NS
Comorbidity						
Cardiovascular disease	41 (65.1)	212 (53.8)	1.60 (0.92 – 2.79)	0.095	NS	NS
**Neurological disease**	**21 (33.3)**	**76 (19.3)**	**2.09 (1.17 – 3.74)**	**0.01**	**2.63 (1.32 – 5.24)**	**0.006**
**Malignancy**	**20 (31.7)**	**57 (14.5)**	**2.75 (1.51 – 5.01)**	**0.001**	**3.85 (1.86 – 7.97)**	**<0.001**
Chronic kidney disease	17 (27.0)	58 (14.7)	2.14 (1.15 – 3.99)	0.02	NS	NS

CI = confidence interval; OR = odds ratio; NS = not significant (by backward multivariate regression); OR = odds ratio. *Boldface indicates independent predictors of delayed defervescence, which are statistical significance (P < 0.05) under the multivariate regression model.

Supplemental Table 9. Predictors of delayed defervescence (ED-to-defervescence > 20 days) in febrile and critically ill adults with source-control-required bacteraemia, categorised into the abscess and non-abscess groups*.

Clinical variables	Patient number with delayed defervescence (%)		Univariate analysis		Multivariate analysis	
	Yes	No	OR (95% CI)	*P* value	Adjusted OR (95% CI)	*P* value
Abscess group, n = 196	n = 89	n = 107				
Bacteraemia source						
**Low respiratory tract**	**29 (27.1)**	**13 (14.6)**	**2.17 (1.05 4.49)**	**0.03**	**2.15 (1.02 – 4.53)**	**0.04**
**Liver abscess**	**18 (16.8)**	**32 (36.0)**	**0.36 (0.19 0.70)**	**0.002**	**0.39 (0.20 – 0.78)**	**0.007**
Etiologic pathogen o *Klebsiella pneumoniae*	37 (34.6)	43 (48.3)	0.57 (0.32 – 1.01)	0.05	NS	NS
Fatal comorbidity (McCabe classification)	33 (30.8)	17 (19.1)	1.89 (0.97 3.69)	0.06	NS	NS
**Comorbid liver cirrhosis**	**19 (17.6)**	**6 (6.7)**	**2.99 (1.14 – 7.84)**	**0.02**	**2.91 (1.09 – 7.75)**	**0.03**
Non-abscess group, n = 68	n = 24	n = 44				
Etiologic pathogen of anaerobe	3 (12.5)	1 (2.3)	6.14 (0.60 – 62.67)	0.12	NS	NS
Comorbid malignancy	6 (25.0)	4 (9.1)	3.33 (0.84 – 13.28)	0.15	1.50 (0.08 – 1.20)	0.53

CI = confidence interval; OR = odds ratio; NS = not significant (by backward multivariate regression); OR = odds ratio. *Boldface indicates independent predictors of delayed defervescence, which are statistical significance ($P < 0.05$) under the multivariate regression model.
